# The dynamic duo: how DNA methylation and gene transcription help diatoms thrive in modern oceans

**DOI:** 10.1093/jxb/erad205

**Published:** 2023-08-03

**Authors:** Xin Lin, Leila Tirichine, Xu Zhang

**Affiliations:** State Key Laboratory of Marine Environmental Science, College of Ocean and Earth Sciences, Xiamen University, Xiamen, 361102, PR China; Nantes Université, CNRS, US2B, UMR 6286, F-44000 Nantes, France; State Key Laboratory of Marine Environmental Science, College of Ocean and Earth Sciences, Xiamen University, Xiamen, 361102, PR China

**Keywords:** Climate change, DNA methylation, gene regulation, transposable elements

## Abstract

This article comments on:

**Wan J, Zhou Y, Beardall J, Raven JA, Lin J, Huang J, Lu Y, Liang S, Ye M, Xiao M, Zhao J, Dai X, Xia J, Jin P.** 2023. DNA methylation and gene transcription act cooperatively in driving the adaptation of a marine diatom to global change. *Journal of Experimental Botany***74,** 4259–4276.

**DNA methylation is essential for maintaining genome stability, mediating gene expression, and aiding species in adapting to their environment. Wan *et al*. (2023) measured the changes in phenotypic traits of the model diatom *Phaeodactylum tricornutum* in response to a 2-year exposure to ocean acidification, warming, or both, and analysed the concomitant changes in DNA methylation and transcriptomic patterns. Their study revealed that DNA methylation and gene transcription work in concert to enable unicellular phytoplankton to adapt to dynamic environmental changes.**

Epigenetics was first defined by Conrad Waddington in 1942 and refers to a phenomenon that affects gene expression and the transmission of genetic information without altering the DNA sequence (Deichmann, 2016). The dynamic nature of epigenetics entails a sophisticated interplay among DNA modifications, chromatin architecture, and regulatory proteins that can exert an impact on gene expression and cellular processes (Turner, 2009). Epigenetics plays a crucial role in maintaining genome stability and aiding species in adapting to their environment (Schmid *et al*., 2018; Zhang *et al.*, 2022). Since their discovery in bacteria in 1925 (Fig. 1), DNA methylation marks have been observed in different kingdoms of life (Law and Jacobsen, 2010). The 5mC DNA methylation mark on transposable elements (TEs) (Box 1) is believed to silence the transposition activity of TEs in the genome, thus maintaining genomic stability (Lanciano and Cristofari, 2020).

**Fig. 1.**
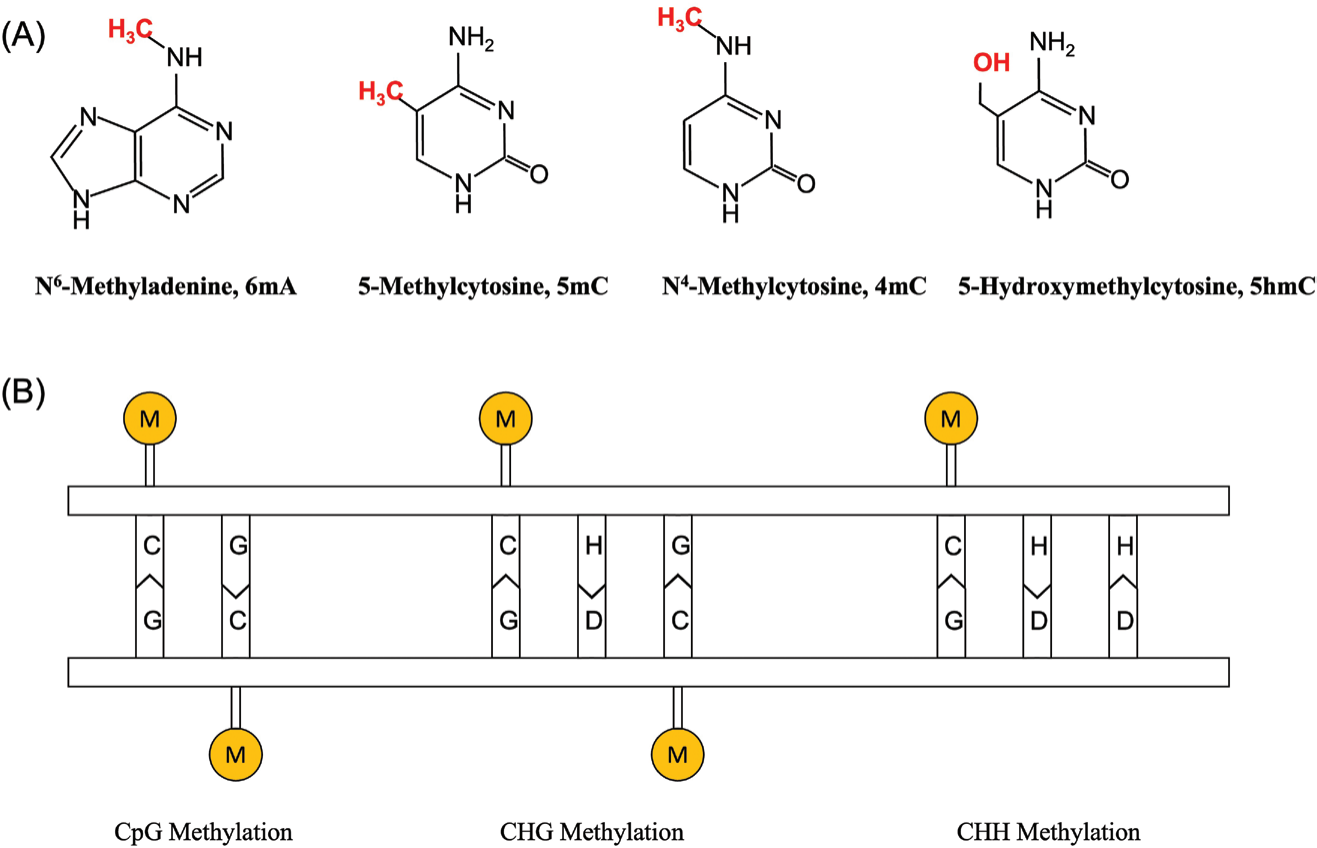
DNA methylation in different contexts. (A) The two most common forms of DNA methylation found in genomes are 5mC and 6mA methylation. In addition, 4mC and 5hmC are less common and can be challenging to detect using conventional methods. In 5mC methylation, a methyl group (CH3) replaces the hydrogen on the fifth carbon atom of cytosine bases, whereas in 6mA methylation, the methyl group replaces the hydrogen on the nitrogen atom of adenine. In addition to regulating gene expression, methylcytosine (5-mC) may be deamino-transformed into thymine (T), causing C–T mutations and producing heritable genetic mutations. (B) While CpG methylation is the predominant form of DNA methylation in mammalian genomes, plant genomes exhibit three distinct methylation contexts CpG, CHG, and CHH (where H represents A, T, or C). The pattern and distribution of DNA methylation vary considerably across the genomes of different organisms, which may be linked to evolutionary processes (Huff and Zilberman, 2014). These methylation patterns have different functions in the regulation of various biological processes (Law and Jacobsen, 2010).

**Box 1. Transposable elements (TEs)**

TEs are a unique class of genomic elements that have the ability to move around within the genome. When TEs become overactive, they may induce changes in gene expression, cause genome expansion, and create instability within the genome. Some TEs contain structural domains that are similar to those found in retroviruses. This similarity suggests that TEs may have evolved from retroviruses. It is worth noting that most eukaryotic and prokaryotic genomes contain a significant number of TEs. The majority of these TEs have lost their transposable function, rendering them inactive. Nevertheless, some TEs, such as long terminal repeat retrotransposons (LTR-RTs), remain active and can have a significant impact on gene expression, phenotypic changes, and even environmental adaptations within a species (Lanciano and Cristofari, 2020).

Taxonomically categorized as Stramenopiles, diatoms hold a significant ecological importance, contributing ~20% to the earth’s primary production (Tirichine *et al.*, 2017). From an evolutionary perspective, diatoms represent the lineage that emerged through secondary symbiosis, distinguishing them from higher plants and green algae that arose from primary symbiosis. Diatoms’ ecological success is remarkable, with epigenetic mechanisms emerging as a plausible factor contributing to their adaptability. This stems from the relatively slow rate of standing stock DNA sequence variations which may not be sufficient to accommodate the rapid and highly dynamic environmental changes that diatoms face (Tirichine *et al.*, 2017). The methylome of marine phytoplankton, specifically the diatom *P. tricornutum*, is a major focus of research and represents a significant milestone in the field. Only 5% of the genome of *P. tricornutum* is methylated, and most methylated regions are rich in TEs, with a prevalence of CG methylation (Veluchamy *et al.*, 2013). In general, DNA methylation levels were found to be low in diatoms, with the exception of *Cyclotella cryptica* (Traller *et al.*, 2016). The sequencing of additional diatom methylomes presents a promising avenue for exploring the potential range of DNA methylation patterns that may exist within this group. Although only a very limited proportion of genes were found to be methylated in CG, CHG, and CHH contexts, gene methylation has been shown to correlate with gene expression regulation in response to nitrate starvation (Veluchamy *et al.*, 2015).

Ocean acidification and global warming are widely recognized as two of the most alarming environmental issues. The importance of DNA methylation in response to short-term ocean acidification and warming in marine organisms has been recently reported (Liew *et al.*, 2018; Lee *et al.*, 2022). Although DNA methylation has been shown to play essential roles in facilitating evolutionary adaptation in the green alga *Chlamydomonas* (Kronholm *et al*., 2017) and the cyanobacterium *Trichodesmium* (Walworth *et al*., 2021), studies on the functions of DNA methylation in diatoms in adaptation to ocean acidification and warming are still very limited. Considering the fundamental ecological significance of diatoms as primary producers and key players in oceanic biogeochemical cycles, deciphering the roles of DNA methylation in their adaptative response to global change is essential.

## The role of DNA methylation in gene expression regulation and adaptation to long-term global changes

In order to investigate the roles of DNA methylation in long-term global change, Wan *et al*. (2023) grew *P. tricornutum* under ocean acidification and/or warming for 2 years. The authors measured the phenotypic trait changes of the diatom with the changes in DNA methylation and transcriptomics, established a link across different levels of biological organization, and tried to bridge the gap between omics and global biogeochemical cycles. The study contributes to a better understanding of epigenetic regulation in the adaptation to climate change in unicellular eukaryotic phytoplankton. The observed DNA methylation levels in *P. tricornutum* were consistent with previous studies reporting a low level of DNA methylation (Veluchamy *et al.*, 2013, 2015; Huff and Zilberman, 2014). Based on their investigation, the authors have postulated that DNA methylation even at low levels may operate in concert with gene transcription to facilitate the long-term adaptive responses of *P. tricornutum* to global changes. However, this is based on only a small proportion of genes (<500 out of 12 233 genes) with methylation. Furthermore, the authors could not conclude through correlation analysis that the occurrence of DNA on the gene body is the causal factor for the transcriptional regulation of genes. It is interesting that DNA methylation in the CHG context was found to be positively correlated with gene expression. The authors reported the same positive correlation for the CHH context, although not uniformly across all conditions. However, it is unclear what specific DNA methylation patterns underlie these correlations. A positive correlation has been observed between DNA methylation in the CHH context at distal promoter regions and gene expression in rice (Zemach *et al.*, 2010a, b). As indicated by the authors, only a minority of differentially expressed genes were located in differentially methylated regions. The study by Veluchamy *et al*. (2015) revealed that DNA methylation levels were positively correlated with a notable up-regulation in the expression of 48 genes in response to short-term nitrate starvation. The available data suggest that DNA methylation is involved in both the short-term acclimation and long-term adaptation of *P. tricornutum* to environmental stimuli. Despite recent advances, the mechanisms by which DNA methylation influence gene transcription during short-term acclimation and long-term adaptation in diatoms remain poorly understood. It has been found that DNA methylation levels are not universally low across all diatom species, thereby adding complexity to the situation. Moreover, the functional implications of DNA methylation in other non-model diatoms which show varying DNA methylation levels in response to environmental fluctuations remain largely unknown. Given the interspecies variations in DNA methylation levels and patterns, it is possible that the roles of DNA methylation may differ across different diatom species and need further investigation.

Previous studies have shown that CHH and CHG contexts of DNA methylation may play a more crucial role in the regulation of genes and TEs (Lin *et al.*, 2017; Domb *et al*., 2020). The authors observed that CHH and CHG methylation was more dynamic and responsive to environmental changes than CG methylation. Moreover, their finding revealed a positive association between CHH methylation within the gene body and gene expression in *P. tricornutum*. Collectively, these findings suggest that different contexts of DNA methylation may have different functions in response to environmental stimuli in diatoms, which deserves further investigation.

## The correlation between DNA methylation and TE expression is to be explored

The authors have comprehensively analysed the correlation between gene expression and DNA methylation in different DNA methylation contexts. Nonetheless, the relationship between TE expression and DNA methylation on TEs was not analysed and discussed. The bursts of TEs are considered as an evolutionary driving force, and the changes of DNA methylation on TEs under a long-term global change scenario may potentially influence genome stability (Lanciano and Cristofari, 2020). In *P. tricornutum*, the primary targets of DNA methylation are TEs (Hoguin *et al.*, 2023); therefore, understanding the correlation between DNA methylation changes in TEs and their impact on TE expression and genome stability is crucial for deciphering the epigenetic regulatory mechanisms underlying adaptation to climate change. However, the multicopy and highly repetitive nature of transposons in the genome poses significant challenges to transposon-related analyses in two primary ways. Firstly, the short fragment sizes produced by second-generation genome sequencing technology hinder the assembly of TEs. Secondly, the quantitative analysis of transposon expression is limited by the presence of multiple copies of transposons in the genome and the difficulty in precisely determining the length of the transposon expression region. Therefore, novel strategies and technologies are needed to enhance the resolution and precision of transposon-related analyses. With the advent of long-read sequencing techniques, such as Nanopore and PacBio, the annotation and expression analysis of TEs can be improved, which may lead to a more nuanced and comprehensive understanding of the specific roles that TEs play in response to global changes (Lanciano and Cristofari, 2020).

## Embracing single-cell sequencing for a better understanding of epigenetic regulation in response to global change in unicellular phytoplankton

As shown in this and previous studies, the RNA-seq and whole-genome bisulfite sequencing (WGBS) analyses were based on the ‘cell population’ for unicellular organisms, overlooking the variations among individual cells. Therefore, the potential diversity of individual cells in response to environmental changes was homogenized based on the current sequencing strategy. The work presented by Wan *et al*. (2023) is a very good starting point for understanding DNA methylation and gene expression in general, but single-cell approaches may yield even more nuanced results and insight. With the advent of rapid development of single-cell sequencing technology, including single-cell genomic, epigenomic, and transcriptomic technology, and the integration of multilayered datasets (Lee *et al.*, 2020), we may be able to better detect the variations of individual cells in response to global changes in the future.
